# Supplementary material for: Are carbon emissions trading and green financial instruments synergistic? -Comprehensive quantitative research based on content analysis
Source: PLoS One. 2024 Mar 7;19(3):e0298601. doi: 10.1371/journal.pone.0298601 (PMC10919601; doi:10.1371/journal.pone.0298601)
Supplement: S1 Data — (ZIP) [file pone.0298601.s001.zip › Data/地级市Z_F工作报告.zip!/║■▒▒/╛ú├┼╩╨/└┤╫╘╣1⁄2╓┌║┼í╛┬φ┐╦╩2╛▌═°í┐_2018.docx]

各位代表：

现在，我代表市人民政府向大会作工作报告，请予审议，并请市政协各位委员和其他列席人员提出意见。

一、2017年工作回顾

刚刚过去的一年，是荆门奋力建设湖北区域性增长极、实现在湖北中部崛起的起步之年。一年来，我们认真贯彻落实党的十九大和省第十一次党代会精神，在省委、省政府和市委的坚强领导下，在市人大、市政协的监督支持下，紧紧依靠和团结全市人民，务实重行、砥砺奋进，较好地完成了市九届人大一次会议确定的目标任务。全年实现地区生产总值1664.17亿元，同比增长7.5%；规模以上工业增加值同比增长7.6%；固定资产投资1790.28亿元，同比增长16.9%；社会消费品零售总额688.08亿元，同比增长12%；实际利用外资41935万美元，同比增长11.4%；出口总额95061万美元，同比增长12.1%；地方一般公共预算收入突破百亿大关，可比增长12.3%；城镇常住居民人均可支配收入31317元，同比增长8.3%；农村常住居民人均可支配收入17167元，同比增长8.6%；居民消费价格涨幅1.6%；城镇登记失业率3.08%；人口自然增长率5.36‰；节能减排完成省定目标。

（一）招商引资和项目建设取得新成效。出台推动创新发展和农业招商引资两个“十项政策”，通过重资产招商引进项目210个，聘请11名招商顾问，举办第三届楚商大会荆门市招商对接会、龙泉中学校友座谈会、“四化同步”示范带招商对接会等专场招商活动，瑞孚信高端原料药、沈阳机床、诗尼曼家居等92个投资10亿元以上重大项目和27家总部企业落户。围绕七大主导产业，建立健全重大项目储备库和建设库，精准策划亿元以上项目800个。创新和完善项目服务机制，实施亿元以上项目938个，同比增加156个，完成投资1265亿元，同比增长27%，南方农机装备产业园、万华秸秆综合利用、弘润特种汽车玻璃原片、雷一重工等380个亿元以上项目开工建设，金泉新材料二区一期、百杰瑞锂电材料一期、泰富重装等339个亿元以上项目建成投产。

（二）传统产业改造和新兴产业培育迈出新步伐。工业经济总量继续领跑全省同类城市，全市新增规模工业企业100家。推进“百企技改行动”“标准与质量提升行动”，实施荆门石化油品质量升级等126个重点技改项目，完成技改投资326亿元，同比增长35%。战略性新兴产业产值同比增长20.8%，初步形成了通用航空、新能源汽车等全产业链条，泊鹭“海王”水陆两栖飞机下线首飞，长丰猎豹整车即将下线，我市成为全国首批、全省唯一的通用航空产业综合示范区。全市新增高新技术企业57家，高新技术产业增加值达到220亿元。20个特色产业集聚区初具规模，县域经济发展模式被省委、省政府推广。加快发展现代服务业，荆门国际内陆港、保税物流中心（B型）启动建设，爱飞客文化展示中心、汇源农谷体验园等投入运营，实现旅游综合收入165亿元，同比增长12.4%。

（三）现代农业发展和农村综合改革彰显新优势。深入推进农业供给侧结构性改革，农业现代化水平监测得分76.6分，农产品加工业总产值与农业总产值之比提高到3.7：1。大力发展高效生态农业，总面积突破135万亩。新增新型农业经营主体1024家、省级以上农业产业化龙头企业13家。建成国家级农民“双创”园区7个、省级现代农业产业园8个、三产融合发展示范区5个、田园综合体5个、农村电子商务服务站1210个。划定永久基本农田548.5万亩，建设高标准农田66万亩，改善灌溉面积30万亩，主要农作物耕种收综合机械化水平达到78.9%，秸秆“五化”综合利用率达到93%。土地按户连片耕种连续两年写入中央一号文件，荣登首届中国“三农”创新榜，全市按户连片耕种面积达到247.2万亩，总体连片率达到75.6%。农村土地“三权”分置改革、农村集体资产股份权能改革、农村承包土地经营权抵押贷款改革、农业水价综合改革、国有林场改革经验在全国、全省推广，为“三农”发展注入了新动能、增添了新活力。面对持续降雨和汉江秋汛的双重考验，全市上下众志成城，全力以赴抗灾救灾，确保了不溃一堤、不倒一坝、不损一站（闸）；对中稻因灾损失实行财政补贴，积极开展抢排、抢收、抢种，最大限度地减少了灾害损失。

（四）新型城镇化建设和城乡绿色发展呈现新格局。成功举办2017中国新型城市论坛荆门峰会，我市跻身国家卫生城市行列，被确定为国家城市“双修”试点城市和装配式建筑示范城市。全市户籍人口城镇化率达到37%。实施中心城区重大城建项目399个，荆门剧院等“十大公用工程”建设加快推进，绿色生态科技产业城会展中心、双喜停车场及生态修复工程、白云大道（中段）综合改造等一批重点城建项目竣工，荆门城市大数据中心投入运营，漳河机场获得通用机场使用许可证，荆门主城区三环线建成通车。扎实推进荆钟、荆京、荆沙三条“四化同步”示范带建设，共实施重大支撑性项目71个，总投资96.8亿元。全域推行河湖长制，全面关停禁养区内养殖场，天门河等重点流域水质稳步提升，空气环境质量改善幅度居全省前列，中央环保督查反馈问题得到有力整改。扎实推进国土资源节约集约示范创建，地质灾害防治“四位一体”网格化管理成效显着。以优异成绩通过国家节能减排财政政策综合示范市验收，京山荣获“国家生态文明建设示范县”称号，钟祥获评“全国森林旅游示范县”，屈家岭成功创建“国家现代农业庄园”“中国农业公园”。特色小镇和美丽乡村建设深入推进，漳河爱飞客小镇、京山网球小镇成为全国首批运动休闲特色小镇，京山马岭村和钟祥南庄村、彭墩村分别入选“中国美丽乡村百佳范例”和“全国美丽乡村示范村”、“中国名村”。

（五）供给侧结构性改革和重要领域改革实现新突破。全面落实“三去一降一补”任务，关停小煤矿、小磷矿、小石膏矿19家，完成企业兼并重组项目30个，消化库存商品住房283.6万平方米，化解金融机构不良贷款19.5亿元。在全省率先成立项目服务局，编制政府部门权责清单、监管清单和马上办、网上办、一次办“三办”清单，集中兑现惠企资金，推行并联审批、容缺审批、多评合一、联审联验，在荆门高新区开展一般工业项目“零审批”试点工作，我市被评为全国商务综合行政执法体制改革优秀试点城市。商事制度改革纵深推进，企业类市场主体总量同比增长21.9%。市级国有资本投资运营公司改革取得实质性进展，全面完成市直单位所属企业和经济实体脱钩改制、资产划转任务，在全省率先建立集中统一的国有资产监管运营体制。开展金融服务实体经济系列活动，全社会融资额突破400亿元；新增“新三板”挂牌企业6家、四板挂牌企业206家；实施PPP项目30个，落地数居全省首位。成立招才引智局和青年创业者协会，成功举办第二届湖北省海智菁英创新峰会暨海智专家荆门行活动，建成创业创新平台29个，创建国家级孵化器（众创空间）3家。荆门高新区获批全国第二批区域“双创”示范基地，在国家级高新区综合排名中跃升13位。

（六）社会事业发展和社会治理创新再上新台阶。民生支出占地方一般公共预算支出的比重达到80.2%，去年年初确定的民生实事全部办结。实施产业扶贫项目673个，完成易地扶贫搬迁3234户、9602人，新增扶贫小额贷款3亿元，光伏扶贫覆盖所有贫困村，实现46个贫困村出列、29975名贫困人口脱贫。

发放创业担保贷款2.1亿元，扶持创业6050人，新增城镇就业4.6万人。五项社会保险参保净增4.2万人次，城乡低保标准分别提高11.3%、28.2%，长期护理保险改革走在全国前列。新开工保障性安居工程19980套，建成15623套，分配入住13263套。新建和改扩建25所幼儿园，完成39所义务教育学校标准化改造，建成龙泉中学南校、高新学校、望兵石学校、德艺学校南校，荆门技师学院被确定为国家高技能人才培训示范基地。完成6个“四化”乡镇卫生院和182个“五化”村卫生室达标创建，公立医院药品加成全部取消，分级诊疗实现全覆盖，家庭医生签约服务率34.5%，在全省率先统一城乡医保制度，H7N9禽流感、诺如病毒等疫情得到有效防控。强化计生优质服务，全面两孩政策有效落实。改造城乡农贸市场39家，中心城区实现“白条禽”上市。新建标准化城市社区42个、新型农村社区示范点68个。书香荆门建设扎实推进，屈家岭、苏家垄考古遗址公园获国家文物局批准立项，成功举办沙洋江汉运河国际半程马拉松赛、中国（京山）绿林网球·英雄会、全国跳伞锦标赛、市第九届老年人体育健身大会等赛事。解决10万农村居民饮水安全问题，汉西水系连通及城市备用水源工程建成通水。“七五”普法工作实现良好开局，“平安荆门”建设和警务体制机制改革深入推进，创新基层社会治理的积分制管理模式全面推广，沙洋捧回全国综治最高奖“长安杯”。安全生产形势总体平稳，一批信访积案得到有效化解，食品药品安全群众满意率进一步提高，社会大局保持和谐稳定。

与此同时，国防动员、国防教育、民兵预备役建设、“双拥”和军民融合工作不断加强，妇女儿童、青少年、老年人、残疾人权益得到有效保障，援疆援恩工作深入开展，哲学社会科学、住房公积金、机关事务、新闻出版、档案、地方志、气象服务、人防建设、统计、物价、移民、保密、科普等工作扎实推进，民族、宗教、外事侨务、红十字、慈善等各项事业健康发展。

一年来，在加快经济社会发展的同时，我们始终坚持依法行政，制定并落实法治政府建设实施方案，建立健全政府学法、规范性文件监督管理、集体议事决策等制度，开展行政执法突出问题专项治理；稳步推进地方立法工作，向市人大常委会提交地方性法规议案1件，出台政府规章《荆门市停车设施规划建设和管理办法》；办理人大代表建议169件、政协委员提案414件。始终坚持高效施政，编制政府重点工作任务落实手册，进一步加大政务督查力度。始终坚持从严治政，认真履行全面从严治党主体责任，推动“两学一做”学习教育常态化制度化，切实加强财政监督、审计监督和行政监察，严肃查处各类违纪违法案件。

各位代表，过去一年的成绩来之不易，积累的经验弥足珍贵，这是省委、省政府正确领导、亲切关怀的结果，是市委统揽全局、科学决策的结果，是全市上下团结奋斗、竞进作为的结果。在此，我代表市人民政府，向全市人民，向人大代表、政协委员，向各民主党派、工商联、人民团体、无党派人士、离退休老同志，向驻荆部队、武警官兵，向关心支持荆门发展的社会各界人士，表示衷心的感谢并致以崇高的敬意！

二、2018年工作安排

各位代表，党的十九大确立了习近平新时代中国特色社会主义思想的历史地位，提出了新时代坚持和发展中国特色社会主义的基本方略，确定了决胜全面建成小康社会、开启全面建设社会主义现代化国家新征程的目标。我们一定要把思想和行动统一到党的十九大精神上来，把智慧和力量凝聚到中央和省委、市委确定的目标任务上来，推动各项战略部署在政府系统落地落实。

——牢牢把握社会主要矛盾的历史性变化，把解决发展不平衡不充分的问题作为根本任务。我国社会主要矛盾已经转化为人民日益增长的美好生活需要和不平衡不充分的发展之间的矛盾。发展不平衡不充分是荆门的基本市情、最大实际，我们要深刻认识荆门发展的阶段性特征，准确研判重大机遇叠加期、转型升级攻关期、建成增长极决战期的“三期”形势，看到经济总量不大、重大项目支撑不够的差距，正视产业结构不优、竞争力不强、新旧动能转换任务艰巨的压力，直面资源环境制约日益突出、生态质量有待提升的难题，聚焦交通物流等基础设施建设滞后、民生保障水平有待提高的短板，紧盯政府系统少数干部能力不足而不能为、激情不足而不想为、担当不足而不敢为的问题，进一步推进治理体系和治理能力现代化，进一步提升发展质量和效益，更好地满足全市人民在经济、政治、文化、社会、生态等方面日益增长的美好生活需要。

——牢牢把握建设现代化经济体系的战略部署，把实现高质量发展作为根本要求。发展是党执政兴国的第一要务。我们要认真贯彻习近平新时代中国特色社会主义经济思想，积极适应经济由高速增长转向高质量发展的新形势，把有质量的速度和有速度的质量有机统一起来，加快推动质量、效率、动力变革，聚焦聚力实体经济发展，培育一批在全国有竞争力、影响力的特色产业集群；聚焦聚力国家创新型城市建设，着力把荆门建设成为中部地区重要的创新之城、创客之乡；聚焦聚力乡村振兴战略，争当全省农业现代化的排头兵；聚焦聚力县域经济发展，形成错位发展、多点支撑的新格局；聚焦聚力供给侧结构性改革和重要领域改革，打造湖北全面深化改革先行区、示范区，不断增强经济创新力和竞争力。

——牢牢把握荆门新时代发展的神圣使命，把加快建成湖北区域性增长极作为首要目标。省第十一次党代会明确要求荆门建设成为湖北区域性增长极，这是省委对荆门的厚望和重托，是荆门新时代发展的职责和使命。我们要努力建设经济发展增长极，确保主要经济指标增幅高于全省平均水平、领跑全省同类城市；努力建设城乡融合发展增长极，切实走在国家新型城镇化综合试点地区前列；努力建设绿色发展增长极，坚持生态立市不动摇，积极建设美丽荆门；努力建设开放发展增长极，对接“一带一路”、长江经济带和中国（湖北）自贸区建设，着力构建对外开放大平台、大通道、大方阵，在湖北“建成支点、走在前列”历史进程中谱写壮丽的荆门篇章。

——牢牢把握以人民为中心的发展思想，把提高群众的幸福感、获得感、安全感作为根本追求。人民对美好生活的向往，就是我们的奋斗目标。我们要多办顺民意、纾民怨、解民忧、增民利的好事、实事，在打好防范化解重大风险、精准脱贫、污染防治的攻坚战上取得扎实进展，在幼有所育、学有所教、劳有所得、病有所医、老有所养、住有所居、弱有所扶上下足功夫，让改革发展成果更多更公平地惠及全市人民。

各位代表，新时代赋予新使命，新使命呼唤新作为。2018年是深入贯彻落实党的十九大精神的开局之年，是实施“十三五”规划的承前启后之年，是决胜全面建成小康社会的关键之年。今年政府工作的总体要求是：全面贯彻党的十九大精神，以习近平新时代中国特色社会主义思想为指引，认真落实省第十一次党代会、市第八次党代会的决策部署，坚持稳中求进工作总基调，坚持以供给侧结构性改革为主线，认真践行新发展理念，落实高质量发展要求，遵循生态立市、产业强市、资本兴市、创新活市路径，更加注重产业转型升级，更加注重创新驱动发展，更加注重提升城市能级，更加注重生态文明建设，更加注重增进民生福祉，为实现全面小康、全面建设湖北区域性增长极奠定坚实基础。

主要预期目标是：地区生产总值增长8%；规模以上工业增加值增长8%；固定资产投资增长13.5%；社会消费品零售总额增长11.5%；地方一般公共预算收入增长9%；实际利用外资增长8%；出口总额增长9%左右；居民消费价格涨幅控制在省定目标以内；城镇登记失业率控制在4.5%以内；人口自然增长率稳定在5.5‰左右；城乡常住居民人均可支配收入增幅与经济增长基本同步；节能减排完成省定目标。重点抓好以下工作：

（一）坚定不移扩大有效投资

以更大力度推进招商引资。聚焦工业、聚焦主导产业、聚焦先进制造业，引进10亿元以上重大产业项目50个以上，招商引资实际到位资金1200亿元以上。扎实推进产业链招商，进一步强化专班招商，创新委托招商、以商招商、股权招商、科技招商、依托商会和基金公司招商等方式，按照“为凤筑巢”要求，提高重资产招商质效。突出投资强度、税收额度、链条长度、环保程度、科技高度，加强对注册率、开工率、到资率、投产率的督办考核。

以更大力度推进项目建设。坚持定时、定项、定向调度，完善重大项目路线图管理、“三级包联”、晾晒拉练和“六有”机制，推动项目快落地、快建设、快投产。统筹推进900个亿元以上项目建设，着力实施一批重大产业项目，开工建设修正药业（荆门）医药产业园、东方雨虹防水材料、玲珑子午线轮胎等项目，加快金泉锂离子电池、奥美（荆门）医用非织造制品等项目建设，推动长丰猎豹汽车、国安新能源汽车零部件、万华秸秆综合利用等项目建成投产；实施一批重大交通基础设施项目，扎实做好襄荆宜高铁、冷水机场改扩建等项目前期工作，开工建设荆荆客专，加快推进蒙华铁路、枣潜高速公路、沙洋汉江二桥建设及G207等15条国省道改造升级工程，确保中心城区汽车客运南北站、钟祥汉江二桥等项目竣工运行，着力构建铁、水、公、空多式联运的综合交通运输体系；实施一批重大水利设施项目，大力推进碾盘山水利水电枢纽工程建设，完成汉江堤防综合整治年度任务，建成钟祥南湖、沙洋马良大型排涝泵站。

以更大力度创优营商环境。编制惠企政策兑现服务指南，建立政策兑现“绿色通道”，实行一窗受理、一站办结。加大闲置低效用地清理整治力度，优先保障骨干企业发展需求；加强产业工人队伍建设，实施“荆门工匠”行动计划，积极推行现代学徒制，为企业提供定向招工、订单培训等服务；大力开展“银企手拉手”活动，构建常态化政银企对接机制，切实缓解实体经济企业融资难、融资贵问题。充分激发和保护企业家精神，实施民营企业家素质提升工程，健全领导干部经常联系民营企业、商会和民营企业政策联络员制度，持续开展“千名干部进千企、创优服务促发展”活动，着力构建“亲”“清”新型政商关系。出台新民营经济发展规划，实施“名家、名企、名品”培育工程，确保民间制造业投资占制造业投资的比重高于85%。

有项目才有发展，有大项目才有大发展。我们要始终把招商引资作为“一号工程”，把项目建设作为“第一位任务”，充分发挥投资对优化供给结构的关键性作用，筑牢荆门科学发展、跨越式发展的硬支撑！

（二）坚定不移加快经济结构战略性调整

加快工业经济转型升级。深入实施“百企技改行动”，完成技改投资400亿元以上；加快磷化、农产品加工、建材等产业链垂直整合，加大企业兼并重组力度；推进先进制造技术、信息技术、智能技术深度融合，培育30家智能化工厂和数字化车间，规模以上企业信息技术应用普及率达到55%。擦亮“新能源汽车之都”、“通用航空新城”两张产业名片，培育壮大智能制造装备、再生资源利用与环保、新能源新材料、新一代信息技术、生物医药等产业，战略性新兴产业产值增长20%以上。支持荆门高新区建设国家创新型特色园区，全市新增高新技术企业50家、科技型中小企业200家，高新技术产业增加值占地区生产总值比重达到13%以上，研究与试验发展经费占比达到2.1%。坚持每个主导产业重点培育3家左右龙头企业，支持80家细分领域“隐形冠军”做大做强，新增规模以上工业企业80家、10亿元企业10家、50亿元企业2家，助推新洋丰、格林美跻身百亿企业行列。

加快培育现代服务业。抓好省级服务业综合改革试点和示范区建设，推动生产性服务业向专业化和价值链高端延伸，生活性服务业向精细化和高品质转变。优化专业市场布局，加快传化公路港（二期）、汉江石牌港等重点项目建设，抓好龙山脊CBD等商圈建设，引进和培育4A级以上物流企业2家，新增限上企业50家。推进大健康产业园建设，新建1—2家中高端医养结合体。实施全域旅游和乡村旅游提升行动，举办2018荆门爱飞客飞行大会，推进圣境山旅游综合开发等项目建设，支持明显陵创5A级景区，实现旅游收入188亿元以上。加快实施国昊文化创意产业园、鳄鱼小镇等重点项目，突破性发展现代设计、动漫创意、文化装备等产业。积极培育数字经济、分享经济等新业态。

加快发展县域经济。坚持全要素、全产业链、全地域谋划、布局和发展县域经济，进一步明确各地产业定位，支持京山智能制造和农机装备制造、沙洋临港装备和新型建材、钟祥数控机床和长寿食品、东宝绿色建材和电子信息、荆门高新区精细化工和新能源汽车、漳河通用航空、屈家岭生态农产品加工、大柴湖汽车电子和精品花卉等产业发展，形成更多集聚度高、成长性好的块状经济体。

经济结构战略性调整，是荆门产业发展迈向中高端的必由之路。我们必须坚持质量第一、效益优先，着力打造荆门经济“升级版”，努力实现更有质量、更有效益、更加公平、更可持续的发展！

（三）坚定不移实施乡村振兴战略

大力推进“三乡工程”。实施能人回乡工程，推广“能人当家”经验，鼓励引导创业有成的能人返乡创办实业，促进农民就业，增加农民经营性收入。实施企业兴乡工程，鼓励引导有社会责任感、有经济实力的企业到农村投资兴业，利用农村资源，推动整村股份合作开发，带领农民增收致富，增加农民工资性收入。实施市民下乡工程，鼓励引导市民下乡过田园生活，长期租用农村空闲农房和农地资源，增加农民财产性收入。

大力推进农村一二三产业融合发展。坚持最严格的耕地保护制度，实施藏粮于地、藏粮于技战略，加快划定和建设粮食生产功能区、重要农产品生产保护区和特色农产品优势区，建设高标准农田66.4万亩，改善灌溉面积30万亩，主要农作物耕种收综合机械化水平达到80%。坚持质量兴农、绿色兴农，推广高效种养模式140万亩，创建40个农牧结合生态养殖示范场、8个水产健康养殖示范场，新认证“三品一标”产品30个，农产品质量安全例行抽检合格率达到97.5%以上。实施“三园一体”建设提升工程，加大农业产业化龙头企业培育和引进力度，加快建设京山桥米和食用菌、沙洋“双低”油菜和水产品、钟祥休闲食品等农产品精深加工集聚区；推动农业与文化、旅游、健康养老深度融合，打造屈家岭农耕文化产业园、彭墩乡村文化体验园等生态旅游基地，建成20个休闲农业示范园，提升国家现代农业示范区和农业科技园区建设水平。积极开发农业多种功能，发展农村电商、农业科普、农业旅游等农村新经济新业态。抓好钟祥市国家农村产业融合发展试点工作。

大力推进“四化同步”示范带建设。支持荆钟示范带打造成为全国转变农业发展方式示范区、中国农谷三产融合发展核心区，荆京示范带打造成为农业科技创新中心、农耕文化旅游区，荆沙示范带打造成为高效生态农业展示区。切实抓好彭墩长寿食品产业园、汇源生态产业园、中国农谷生态农产品加工产业园、洪森·花果谷生态休闲农业观光园、G207改扩建配套慢行生态绿道等重大项目建设。坚持“三生”融合、产城融合，加快建设漳河爱飞客小镇、石牌豆腐小镇、大柴湖花卉小镇、孙桥对节白蜡艺术小镇、太子森林小镇等特色小镇。加大美丽宜居乡村建设力度，创建15个省级、100个市级美丽宜居示范村。

大力推进农业农村改革。深化农村土地“三权”分置改革，探索土地流转股权化模式。建立工商资本开展土地流转审核机制。完成农村集体资产股份权能改革任务。构建新型农业经营主体政策支持体系，强化与农民利益联结。创新农村金融服务机制，组建涉农信贷担保机构，发挥好农业贷款风险补偿基金作用，引导金融机构加大涉农信贷投放力度。健全农村社会化服务机制，深化供销合作社综合改革，推行政府购买农业公益性服务模式。深化农业水价综合改革。扎实推进农垦改革。

实现全面小康，绝不能一头是发达的工业和繁荣的城市，另一头是传统的农业和落后的农村。我们要大力实施乡村振兴战略，持续推进中国农谷建设和柴湖振兴发展，努力使农业成为有奔头的产业、使农民成为有吸引力的职业、使农村成为安居乐业的美丽家园！

（四）坚定不移建设现代化精致城市

进一步提升城市规划水平。突出规划引领，认真谋划城市“成长坐标”，编制完成荆门2049城市远景发展战略规划，推进京山、沙洋、钟祥总体规划修改工作。加强城市重点区域控规修改和城市设计，编制海绵城市、中心城区“双修”等专项规划。落实历史文化名城保护规划，推动历史街区保护和更新。强化对规划编制和实施的监管，坚持“开门”抓规划，增强规划的科学性、权威性、严肃性。

进一步提升城市建设水平。注重传承历史和文化记忆，将文化元素融入城市建设，提升城市内涵和品质。加大老城区“双修”力度，着力实施“两山”改造工程，推动竹皮河实现水清、水美、水生态及景观步道连通；加快推进阳光大道、荆东大道等道路改造项目，完成白云大道（南段）综合整治和三条高速公路出入口景观提升工程建设，不断改善市民出行条件。积极拓展新城区，规划建设高铁新城，加快绿色生态科技产业城等项目建设，推进凤凰湖湿地公园二期、天鹅池公园、杨柳湖湿地保护等项目实施，完成环库绿道建设。不断完善功能区，大力推动长宁新城、温泉新城及未来科技城建设，加快打通荆山大道、龙井大道、华科路等主次干道，促进产城融合发展。支持京山深化国家生态文明建设示范县建设、沙洋打造滨江园林城市和生态宜居城市、钟祥创建国家园林城市和国家卫生城市。

进一步提升城市管理水平。巩固和扩大创卫成果，积极创建全国文明城市。坚持精细管理，修订完善城市管理标准，抓好深度保洁，打造干净城市。实施新型智慧城市总体规划，推进智慧城管、智慧交通等试点示范建设。配套完善停车设施，打通城市“微循环”，加快天龙公共人防工程等项目建设，不断缓解交通压力。大力开展城市顽疾整治，加强市民素质教育，着力解决超载超限、运输抛撒、经营秩序、背街小巷卫生等问题。严厉打击违建行为，加快处置存量违建。

人民城市人民建，建好城市为人民。我们要坚持“雅、朴、净、畅、宁”的导向，推动城市紧凑集约、高效绿色发展，努力让人民群众在城市生活得更方便、更舒心、更美好！

（五）坚定不移打造美丽荆门

着力构建生态安全格局。落实长江经济带“共抓大保护、不搞大开发”要求，完成“一带两屏四网六廊”生态格局规划编制。划定生态保护红线、永久基本农田、城镇开发边界三条控制线，筑牢生态安全屏障。大力开展生态市、县、镇、村“四级同创”，积极创建国家环保模范城市。

着力推动绿色低碳循环发展。加快传统产业绿色改造，发展壮大节能环保产业、清洁生产产业、清洁能源产业。强力推进节能减排，在漳河新区等生态环境优良区域，实施近零碳排放区示范工程。深入推进国土领域供给侧结构性改革和节约集约示范创建。加大国家循环经济示范市建设力度，打造荆门化工、格林美再生资源回收利用、东宝农作物废弃物综合利用等循环经济产业园区。推进静脉产业园建设，开展垃圾分类试点工作，加快城乡垃圾处置减量化、资源化、无害化步伐。

着力加强生态环境综合治理。深入实施生态环境治理“十大工程”，促进生态环境持续改善。打好蓝天保卫战，坚持抑尘、压煤、控车、禁烧、提标、增绿并举，确保中心城区PM10、PM2.5年均浓度分别控制在86、55.7微克/立方米以内，空气质量优良天数比例达到78%以上。打好碧水保卫战，严格落实河湖长制，大力推进防洪水、排涝水、治污水、保供水“四水共治”，像保护生命一样保护漳河、温峡、惠亭等饮用水源地，强力推进汉江、天门河、竹皮河、长湖等重点流域综合整治，确保天门河稳定在四类水质，并逐步达到三类水质。建立全市土壤污染防治数据库，完成钟祥局部土壤重金属污染治理、重金属污染农田土壤修复与治理试点示范工程。加强地质灾害防治，实施矿山地质环境恢复治理工程。

着力建立生态文明建设长效机制。创新生态环境保护和治理投入模式，推行环境污染第三方治理，建立市场化、多元化生态补偿机制和生态环境损害赔偿机制，完善用能权、用水权、排污权、碳排放权交易制度。全面落实领导干部自然资源资产审计、自然资源资产负债表编制制度。贯彻《荆门市生态环境保护条例》《荆门市生态环境损害责任追究实施细则》，形成源头严防、过程严管、后果严惩的生态环境硬约束。

现代化是人与自然和谐共生的现代化。我们要牢固树立绿水青山就是金山银山的理念，加快建设美丽荆门，努力打造人与自然生命共同体！

（六）坚定不移推进改革开放

深入推进供给侧结构性改革。坚持用市场化、法治化方式去产能，全面关停煤矿，压减低端肥产能50万吨。建立多主体供应、多渠道保障、购租并举的住房制度，加大棚户区改造货币化安置力度，确保全市商品住房平均去化周期保持在合理区间。引导企业运用股权融资、市场化债转股等手段，积极稳妥降低杠杆率。进一步降低制度性交易成本和生产要素成本，全年为企业减费让利60亿元。进一步加大公共服务、基础设施、生态环保、交通物流、文化产业等领域补短板力度。

扎实推进“放管服”改革。深化相对集中行政许可权改革，完善行政审批局、项目服务局运行机制，推动县（市、区）真正实现事权、人员、平台“三集中”。完善“三办”服务机制和“一站式”集成服务模式，推动“一次办”向“马上办”、“马上办”向“网上办”迈进。持续开展涉企收费和中介服务机构清理整治，建好用好“中介超市”，扩大政府购买中介服务范围，优化中介服务政府采购流程。在工业园区全面推行一般工业项目“零审批”，变先批后建为边建边验、先建后验。全面推行公平竞争审查制度，推动商事注册便利化，抓好“双随机一公开”监管工作。深化“互联网+”政务服务，实现市、县、乡、村四级政务服务事项“一网运行”。继续推进农业、商务、文化、城管等重点行业领域综合执法体制改革，建立跨部门综合执法、跨区域联动联调机制。

积极推进投融资体制机制改革。大力发展普惠金融、绿色金融、科技金融，引导金融机构加大信贷投放力度，新增社会融资400亿元。扩大直接融资规模，加快鄂中生态、博士隆上市步伐，力争6家企业在“新三板”挂牌，全年发行债券50亿元以上。规范产业基金运营管理，以市场化方式拓展基金直投业务，加快基金投资项目落地。聚焦重点地区、重点银行、重点企业，加大金融机构不良贷款化解力度，力争不良贷款率控制在3%以内。深入推进非法集资、恶意逃废债、互联网金融风险、“三角债”清欠等专项整治，妥善处置一批重点金融案件，坚决守住不发生区域性金融风险的底线。

统筹推进其他领域改革。探索农业转移人口市民化新机制，力争常住人口城镇化率达到59%、户籍人口城镇化率达到40%。以加强国有资产监管、完善法人治理结构、优化产权结构为重点，深化国有企业改革。推动市、县国有资本投资运营公司转型发展，在具备条件的市级公司下属企业开展混合所有制改革。抓好驻荆央企、省企及市属国有企业“三供一业”分离移交改革。继续落实积极的财政政策，深化政府采购制度和预算绩效管理改革。进一步规范政府举债融资行为，切实防范政府债务风险。抓好审计管理体制改革，不断提高审计监督实效。深入推进事业单位分类改革，完成事业单位公务用车改革。以实战实用实效为重点，全域推广交巡特警一体化改革。

持续推进对外开放。加强开放枢纽项目建设，推进荆门国际内陆港和保税物流中心（B型）建设。大力实施外贸产业培育工程，扩大加工贸易份额，发展跨境电商、外贸综合服务等外贸新业态。加大外贸骨干企业扶持力度，新增出口企业15家。积极组织企业参加境外展会及国际市场开拓“百展行动”，支持优势企业海外投资、跨国并购。

发展无止境，改革无退路。我们一定要以攻城拔寨、闯关破障的勇气抓好“四梁八柱”性改革，提高经济的外向度，坚定不移地向改革开放要动力、要活力、要红利！

（七）坚定不移保障和改善民生

打好精准脱贫攻坚战。聚焦深度贫困对象、聚焦“两不愁三保障”标准，全面推广“4+N”产业发展模式，开展健康扶贫“三个一批”行动，加大教育扶贫力度，抓好易地扶贫搬迁和危房改造工作，实施深度贫困村提升工程，实现34个贫困村、24632名贫困人口稳定脱贫。强化脱贫攻坚责任，加强督查巡查，保证现行标准下的脱贫质量，切实做到扶贫工作务实、脱贫过程扎实、脱贫结果真实。

千方百计扩大就业创业。实施就业优先战略和积极就业政策，加强困难群体就业援助，做好淘汰落后产能中失业人员再就业工作，支持农村劳动力转移就业、返乡就业。推动大众创业万众创新政策落地，新建“双创”平台5家，发放创业担保贷款2亿元，扶持创业5000人。实施“引智留荆”工程，大力发展人力资源服务业，积极推进人才公寓建设，引导更多大学生在荆创业、更多优秀人才来荆发展。

织密扎牢社会保障网。全面推进养老保险制度改革，完成机关事业单位人员养老保险制度的承接转换，引导城乡居民参加企业职工养老保险。大力开展医保支付改革，实现医保支付网络连通到村。充分发挥失业保险援企稳岗作用。进一步提高城乡低保、大病保险、医疗救助、临时救助和特困供养水平。健全农村留守儿童和妇女、老年人关爱服务体系。进一步做好城镇困难职工解困脱困工作。加快老龄事业和健康产业发展，力争居家养老服务基本覆盖全部城镇社区、老年人互助照料活动中心覆盖60%以上的行政村。发展残疾人事业，加强残疾康复服务。

大力发展社会事业。坚持把教育事业放在优先位置，实施学前教育“第三期行动计划”，推进城乡义务教育一体化发展，加快普及高中阶段教育，大力发展现代职业教育、特殊教育和网络教育，推动荆楚理工学院、荆门职业学院、荆门技师学院更好服务地方发展。规范社会力量兴办教育，促进民办教育健康发展。深化公立医院综合改革，建立现代医院管理制度，完善药品供应保障体系，健全分级诊疗和综合监管制度，加快推进紧密型医联体建设、家庭医生签约服务。落实全面两孩政策，完善生育支持、婴幼儿照护、妇幼健康服务体系，开展“新市民健康城市行”活动，推动计划生育转型发展。全面建设文化小康，完善基层公共文化设施，推进基本公共文化服务标准化、均等化。着力建设书香荆门，加快中国农谷博物馆、屈家岭遗址博物馆建设，完成市博物馆新馆和市图书馆新馆主体工程，加大古遗址、古民居、古村落保护和开发力度。深化新闻出版保障体制改革，大力推进媒体融合发展。大力发展体育产业，广泛开展全民健身活动，支持各县（市、区）打造特色运动休闲小镇。加强新型智库建设。繁荣中国特色哲学社会科学和社会主义文艺。

创新社会治理。深入推进“七五”普法工作，完善公共法律服务体系。建立健全群众诉求表达、权益保障、利益协调和矛盾纠纷化解机制，扎实做好网上群众工作，打造阳光信访、责任信访、法治信访。强化社会治安防控体系建设，依法打击各类违法犯罪行为。推动安全生产领域改革发展，开展“打非治违”专项行动，实现“一无两降”目标。加强食品药品监管，确保群众“舌尖上的安全”。加强村（社区）治理体系建设，全面推行积分制和“小微权力”清单管理，创新基层社会治理，开展新型综合社区建设试点，完成村（居）换届工作。积极创建国家社会信用体系建设示范城市。

牢固树立总体国家安全观，加强国防、人防和民兵预备役建设，推动军民融合深度发展，扎实做好“双拥”工作。开展第四次全国经济普查相关工作。强力推进全国质量强市示范城市建设。进一步做好外事侨务、民族、宗教、援疆援恩、对台、保密、档案、地方志、物价、移民、地震、气象、机关事务等工作。发挥好工会、共青团、妇联、科协、侨联、工商联、残联等群团组织的重要作用。

各位代表，在做好以上工作的同时，我们还将着力办好以下民生实事：

1.为因病致贫的深度贫困对象每人购买300元补充医疗保险，建立健全基本医疗保险、大病保险、医疗救助、补充医疗保险相结合的健康扶贫兜底保障体系。

2.新增城镇就业45500人，组织就业创业培训26000人。

3.解决10万农村居民饮水安全问题。新建及改造389个农村电网配电台区。

4.新开工保障性安居工程11584套，基本建成4129套，分配入住4000套。

5.完成全市乡镇福利院“冬暖工程”和“平安工程”建设。

6.扩大长期护理保险覆盖面，参保人数达到160万人。

7.完成义务教育学校标准化建设和“全面改薄”任务。

8.加快“希望家园”建设，招募大学生志愿者1000名，服务留守儿童6000人以上。

9.实施“一村一名大学生村医”培养计划，2018年免费培养100名临床专业全日制大专学历乡村医生。实现农民体育健身工程全覆盖。

10.推进“厕所革命”，实施农村无害化厕所改造11000所，新建和改扩建城市公共厕所100所。

民生是发展的目的，民心是最大的政治。无论今年财政压力有多大，民生投入只多不少，社会事业支出只增不降，为群众办的实事只加不减，努力让孩子们快乐成长、青年人乐业创业、中年人放心打拼、老年人颐养天年，让全市人民生活得更加幸福、更有尊严！

三、着力建设人民满意的政府

各位代表，政府自身建设永远在路上。我们将持之以恒地加强政府自身建设，让组织放心，让人民群众满意。

（一）旗帜鲜明讲政治。坚持以习近平新时代中国特色社会主义思想武装头脑、指导实践、推动工作，坚决维护以习近平同志为核心的党中央权威和集中统一领导，树牢“四个意识”，增强“四个自信”，做到绝对忠诚于党、绝对忠诚于党的领袖、绝对忠诚于党的事业、绝对忠诚于人民。精心谋划和扎实开展全市政府系统“不忘初心、牢记使命”主题教育，努力使主题教育的过程成为坚定信仰、筑牢理想的过程，成为提升素质、增长本领的过程，成为推动发展、造福人民的过程。坚决贯彻落实党中央国务院、省委省政府和市委的各项决策部署，始终做到在大局中定位、在大局中谋划、在大局中工作，在任何时候、任何情况下都不含糊、不犹豫、不动摇、不打折扣。

（二）抢拼严实强执行。始终把抓落实作为政府工作的生命线和主旋律，着力构建定责、履责、督责、追责一体化的责任链条，努力以政府的落实指数，提升荆门的发展指数、城市的繁荣指数和群众的幸福指数。综合运用常态督查、专项督查、延伸督查、网上督查，加强跟踪问效，构建多层次、全方位、高频率的“大督查”工作格局。强化考评结果运用，对不作为慢作为、怠政懒政的干部公开曝光、从严问责，切实提升政府系统执行力，打通政策落实的“最后一公里”。

（三）攻坚克难勇担当。坚持与时俱进，加强新理论、新知识学习，着力培养政府系统工作人员的专业能力、专业素养、专业精神，做到既有担当的“宽肩膀”、又有成事的真本领。坚持迎难而上，切实做到在政策之内抢着干、政策之外探索干，以敢闯敢试的魄力创新求变。坚持凝心聚力，完善容错纠错、减责免责机制，坚定不移地为改革创新者鼓劲，旗帜鲜明地为真抓实干者撑腰，让想干者有奔头、让敢干者没顾虑、让干成者得重用。

（四）规范用权重法治。自觉接受市人大及其常委会法律监督、工作监督和市政协民主监督，认真办理人大议案、代表建议和政协委员提案，落实重大决策出台前向市人大及其常委会报告制度，广泛听取各民主党派、工商联、无党派人士和社会各方面意见。严格执行重大行政决策法定程序，进一步规范行政执法行为。推进决策公开、执行公开、管理公开、服务公开、结果公开，让政府的一切权力置于法治的阳光之下。

（五）清正廉洁树形象。充分发挥政府党组的“头雁效应”，压实全面从严治党主体责任，始终把纪律挺在前面，把规矩记在心中。认真落实中央八项规定实施细则精神和省、市实施办法，强化对厉行节约、公务接待、职务消费等规定执行情况的常态化监督，切实解决表态多调门高、行动少落实差问题，坚决防范和查处各种隐性、变异的“四风”问题。积极支持监察体制改革试点工作，实现对所有行使公权力的公职人员监察全覆盖。坚持无禁区、全覆盖、零容忍，坚持重遏制、强高压、长震慑，重拳查处各类违纪违法案件和群众身边的“微腐败”问题，让消极腐败现象无处藏身，让清明政府形象深入人心。

各位代表，我们正站在新时代的历史起点上，梦想照亮未来，奋斗成就辉煌。让我们更加紧密地团结在以习近平同志为核心的党中央周围，高举习近平新时代中国特色社会主义思想伟大旗帜，在省委、省政府和市委的坚强领导下，不忘初心、牢记使命，真抓实干、开拓创新，为全面建设湖北区域性增长极、实现在湖北中部崛起而不懈奋斗！
